# An Eco-Friendly Technique: Solvent-Free Microwave Synthesis and Docking Studies of Some New Pyridine Nucleosides and Their Pharmacological Significance

**DOI:** 10.3390/molecules24101969

**Published:** 2019-05-22

**Authors:** Majed Alrobaian, Sana Al Azwari, Amany Belal, Hany A. Eldeab

**Affiliations:** 1 Department of Pharmaceutics and Pharmaceutical Technology, College of Pharmacy, Taif University, Taif 5700, Saudi Arabia; majed.alrobaian@hotmail.co.uk; 2 Department of Information Technology, College of Computers and Information Technology, Taif University, Taif 5700, Saudi Arabia; SANA.ALAZWARI@HOTMAIL.CO.UK; 3 Department of Pharmaceutical Chemistry, College of Pharmacy, Taif University, Taif 5700, Saudi Arabia; abilalmoh1@yahoo.com; 4 Department of Medicinal chemistry, Faculty of Pharmacy, Beni-Suef University, Beni-Suef 1441, Egypt

**Keywords:** green chemistry, microwave synthesis, pyridine galactosides, anticancer activity, antimicrobial, molecular docking

## Abstract

Two series of novel 5-arylazo-3-cyano-2-(2″,3″,4″,6″-tetra-*O*-acetyl-β-d-galacto pyranosyloxy) pyridines and 3-cyano-2-(2″,3″,4″,6″-tetra-*O*-acetyl-β-d-galactopyranosyloxy) pyridines were synthesized in high yields utilizing a microwave-assisted synthesis tool guided by the principles of green chemistry. The chemical structures of the new substances were confirmed on the basis of their elemental analysis and spectroscopic data (FT-IR, 1D, 2D-NMR). Activity against different bacterial strains was studied. The anticancer potential of the new compounds is also discussed. Molecular docking was used as a tool in this research work to get better insight into the possible interactions, affinities, and expected modes of binding of the most promising derivatives of the potential chemotherapeutic target (DHFR).

## 1. Introduction

The production of organic chemicals as reagents or raw substances for many applications, such as pharmaceuticals, manufacturing polymers, artificial fibers, pesticides, paints, and food additives, is continuous. Conventional methods have been employed for decades to synthesize organic chemical substances. There are many harmful situations in terms of safety and health hazards for workers caused by these processes and the disposition of waste. On the other hand, pharmaceutical companies tend to highlight their role in saving lives, with little impact on the manufacturing processes that might have an effect on public health and the environment. “Green Chemistry” expresses a great scientific interest in changing the methodologies of organic synthesis to advance on a small-scale laboratory level and also extend to industrial large-scale manufacturing methods. Green chemistry techniques can help to increase the effectiveness of synthetic methods, to use less toxic solvents, to reduce the steps of synthetic ways, and to reduce waste as far as practically convenient. In this direction, organic synthesis is considered a part of potential sustainable progress. In recent decades, pyridine analogues have been identified as a cornerstone in medicinal chemistry, showing diverse pharmacological actions such as antibacterial [1,2], antifungal [3], anti-inflammatory [4], antiviral [5,6], antitumor [7], and antiplatelet [8,9] properties. In addition, 2-pyridone derivatives are utilized in the production of paints [10], pigments, acid–base indicators, stabilizers for polymers and coatings, and additives for fuels and lubricants [11]. Various pyridine nucleosides have been outlined to have important biological properties [12]. In particular, 4-Amino-3-fluoro-1-(β-d-ribofuranosyl)-2(1*H*)-pyridone **I** inhibits the growth of HL-60 lymphoid leukemia cells with IC_50_ = 1.07 × 10^−5^ M, while a 2′-deoxy analogue of **I** has a potent effect on lymphoid leukemia L1210 cells. In addition, an acetyl derivative of **I** exhibits similar potent activities [13]. Selective inhibitors for the human immunodeficiency virus type 1 reverse transcriptase (HIV-RT) (for example, 3-(4′,7′-Dimethyl benzoxazol-2′-yl)-amino-5-ethyl-6-methyl pyridine-2(1*H*)-one (**II**) and its 4,7-dichloro analogue, which are derivatives of pyridine) have been reported to inhibit the spread of HIV-1 infection by 95% in MT4 cell cultures and were appointed to clinical trials as antiviral agents. In addition, 4-Benzylpyridone (**III**) has been shown to possess potent HIV-1 specific reverse transcriptase inhibitor properties [14].


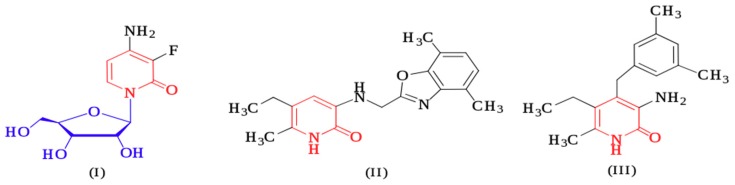


All of these facts prove the importance of pyridine derivatives as promising bioactive agents. In addition, some pyridine-2(1*H*)-one derivatives have been shown to be hopeful antineoplastic active agents for the treatment of cancers [15] and to possess potential activity against different microorganisms [16,17,18]. This guided us to prepare novel pyridine nucleoside analogues in solvent-free medium using microwave irradiation as an economic, fast, ecofriendly, and efficient method for getting these promising candidates.

## 2. Results and Discussion

### 2.1. Chemistry

**Scheme 1:**
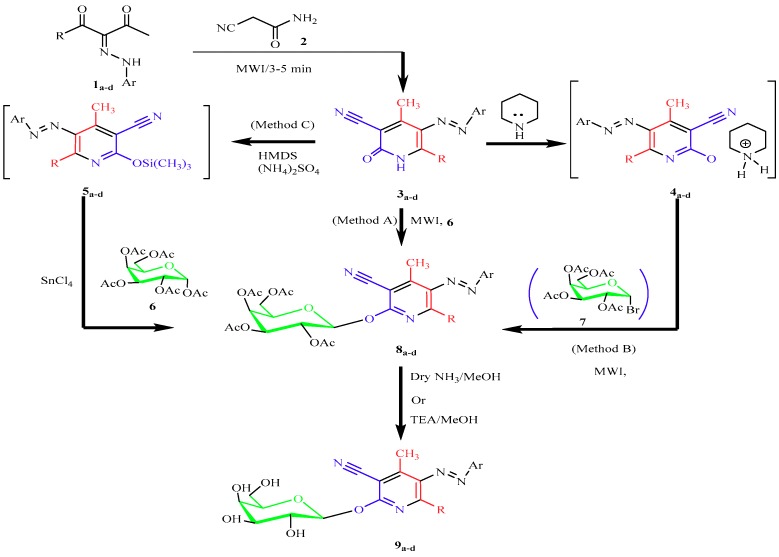
Synthetic pathways of 5-arylazo-3-cyano-2-(2″,3″,4″,6″-tetra-*O*-acetyl-β-d-galactopyranosyloxo)-pyridines (**8_a–d_**).

**Scheme 2:**
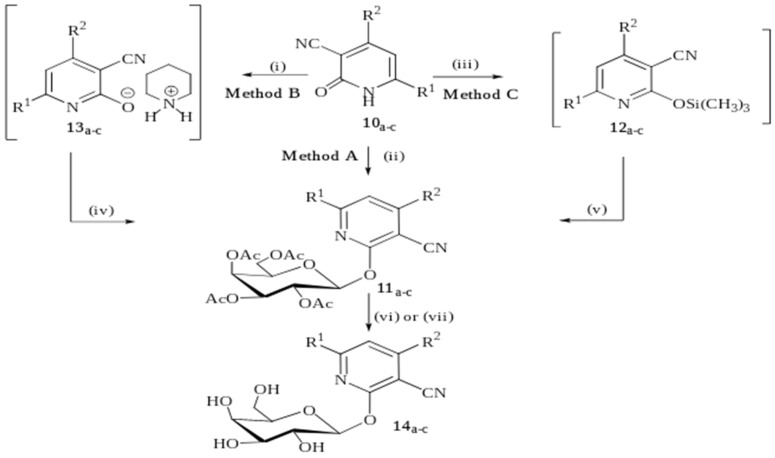
Synthesis of 3-Cyano-2-(2″,3″,4″,6″-tetra-*O*-acetyl-β-d-galactopyranosyloxo)-pyridines (**11_a–c_**). Reagents: (i) Pip., 4 h, 80 °C; (ii) 1″,2″,3″,4″,6″-penta-*O*-acetyl-α-d-galacto-pyranose, silica gel, MW (200 W, 2–3 min); (iii) hexamethyldisilazane, (NH_4_)_2_SO_4_, reflux, 48 h, N_2_, 2″,3″,4″,6″-tetra-O-acetyl-α-D-galactopyranosyl bromide (**7**); (iv) acetone/DMF, MW (200 W, 6–7 min), 1″,2″,3″,4″,6″-penta-O-acetyl-α-d-galactopyranose (**6**), dry MeCN, SnCl_4_, 0–25 °C; (v) TEA/MeOH; (vi) dry NH_3_/MeOH; (vii) TEA/MeOH.

A one-pot microwave synthetic protocol was used to obtain a variety of different pyridine-2(1*H*)-one derivatives (**3_a–d_**) and (**10_a–c_**) [19,20]. In this paper, three different synthetic strategies to construct pyridine galactosides (**8_a–d_**) and (**11_a–c_**) were employed. Microwave synthetic methods (methods A and B) were used as simple, efficient approaches to the preparation of the targeted galactosides **(8_a–d_)** and **(11_a–c_)** in a limited time with an excellent yield. In method A, the solvent-free reaction was conducted using microwave irradiation to enhance the reaction between the routine pyridine-2(1*H*)-ones (**3_a–d_, 10_a–c_)** and 1″,2″,3″,4″,6″-penta-*O*-acetyl-α-d-galactopyranose (**6**) in the presence of a catalyst [21] to afford the 3-cyano-2-(2″,3″,4″,6″-tetra-*O*-acetyl-β-d-galactopyranosyloxy) pyridines (**8_a–d_**) and (**11_a–c_**) in high yields (87–95%). The same galactosides (**8_a–d_**) and (**11_a–c_**) were obtained in better yields utilizing the pyridinium salts (**4_a–d_, 13_a–c_)** and (2″,3″,4″,6″-tetra-*O*-acetyl-α-d-galactopyranosyl bromide) (**7**) (in dry acetone/DMF without any catalyst under microwave irradiation). A classical procedure (C), an expensive and time-consuming approach, was established for nucleosidation via formation of the 2-silyloxypyridine intermediates (**5_a–d_, 12_a–c_**) (Scheme 1 and Scheme 2). Yields and reaction times given in the three synthetic pathways were compared (Table 1 and Table 2). The chemical structures of the resulting galactosides (**8_a–d_**) and (**11_a–c_**) were set up and confirmed on the basis of their elemental analyses and spectral data (LC-MS, IR, UV, 1D- and 2D-NMR). Thus, the analytical data for (**8_c_**) had a molecular formula of C_33_H_31_ClN_4_O_10_, and LC-MS (ionization method) yielded *m*/*z* 679 [M + H]^+^. The ^1^H-NMR (400 MHz, CDCl_3_) spectrum showed the anomeric proton as a doublet at δ = 5.99, *J*_H-1__″__-H2__″_ = 8.3 Hz due to diaxial orientation between the H-1″ and H-2″ protons, indicating a β-configuration and ^4^*C*_1_ conformation. On the other hand, the nine aromatic hydrogen atom signals appeared as a multiplet at δ = 7.60 ppm. ^13^C-NMR was used for further confirmation. However, the results were in agreement with the assigned structure. The nitrile carbon atom appeared at δ 113.8 ppm, while four signals showed at δ 168.3, 169.6, 170.0, and 170.9 ppm, assigned to the four acetoxy carbonyl carbon atoms. The IR spectrum showed bands at υ 2228 and 1757 cm^−1^, indicating the presence of the CN and C=O of acetoxy groups, respectively. On the other hand, no peak corresponding to the amide carbonyl at the pyridine C-2 indicated that the sugar was linked to the pyridine ring through oxygen at C-2, giving the *O*-galactosides as suggested (Scheme 1 and Scheme 2). More specifically, the formation of (**8_c_**) was proven using the phase-sensitive 2D-NOESY spectrum, which revealed the presence of inter-ring cross-peaks between the anomeric proton H-1″ at δ = 5.99 ppm and both H-5″ at δ = 3.94 ppm and H-3″ at δ = 4.26 ppm. Meanwhile, no cross-peak interaction was observed between the anomeric H-1″ at δ = 5.99 ppm and *ortho*-phenyl protons at δ = 7.60 ppm at the pyridine C-6, supporting the formation of *O*-galactosides as the sole isomer and not *N*-glucosides. A long-range ^1^H-^13^C heteronuclear correlation was employed to assign clear structure elucidation, whereas methyl protons at pyridine C-4 (δ = 2.56 ppm) showed strong cross-peak interactions with pyridine C-3 (δ = 94.8 ppm). A weak cross-peak interaction was observed between methyl protons at pyridine C-4 (δ = 2.56 ppm) and carbonitrile carbon (C≡N) (δ 113.8 ppm). The data obtained from 2D-GHMBC strongly agreed with the mechanistic pathway shown in Scheme 1.

Dry ammonia or triethyl amine in methanol methods were applied to convert the protected galactosides into their related free galactosides. Table 3 and Table 4 show the yield comparison between the two methods.

### 2.2. Antimicrobial Activity

**Figure 1:**
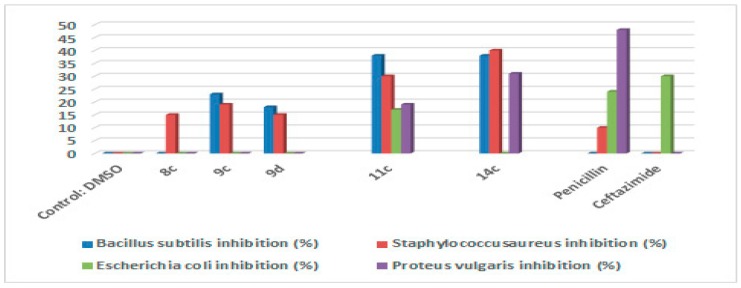
Antimicrobial activity of the active synthesized compounds.

A panel of standard strains of the Institute of Fermentation of Osaka (IFO), namely gram-positive bacteria (*Staphylococcus aureus* IFO 3060 and *Bacillus subtilis* IFO 3007) and gram-negative bacteria (*Escherichia coli* IFO 3301 and *Proteus vulgaris* IFO 3851), were used to investigate the antimicrobial potential of all of the newly synthesized substances (**8_a–d,_ 9_a–d,_ 11_a–c_**, and **14_a–c_**) (30 μg/disc). However, the obtained data of the preliminary antimicrobial screening of the new compounds (**8_a–d,_ 9_a–d,_ 11_a–c_**, and **14_a–c_**) were compared to standard samples of the antibacterial antibiotics penicillin and ceftazidime (30 μg/disc). The results revealed that the obtained substances showed varying degrees of inhibition against the tested microorganisms. New substances (**8_c,_ 9_c,_** and **9_d_**) exhibited a moderate inhibitory effect on the growth of the gram-positive bacteria, while the obtained compounds (**11_c_** and **14_c_**) were effective at inhibiting the growth of both the gram-positive bacteria and the gram-negative bacteria compared to the local samples, as shown in Figure 1. However, the hydroxy and trifluoromethyl groups contributed to raising the antibacterial potential of the new pyridine galactosides against both the gram-positive and gram-negative bacteria strains. 

### 2.3. Anticancer Activity

The newly synthesized compounds (**8_a–d,_ 9_a–d,_ 11_a–c_**, and **14_a–c_**) were tested for their in vitro antitumor activity against lung cancer cells (NCI-H460-Luc2), liver cancer cells (HEPG-2), and breast cancer cells (MDA-MB-231). The newly synthesized compounds exhibited cytotoxic activity against NCI-H460-Luc2 and liver cancer cells (HEPG-2). However, compound (**9_d_**) revealed moderate antitumor activity against both the lung cancer cells NCI-H460-Luc2 (with IC_50_ = 31.2 μM) and the liver cancer cells HEPG-2 (IC_50_ = 51.6 μM) and low activity against the breast cancer cells MDA-MB-231 (IC_50_ = 72.9 μM). The compounds **8_d_**, **11_c_**, and **14_c_** showed moderate activity against NCI-H460-Luc2 and noticeable activity against both the HEPG-2 and MDA-MB-231 cancer cells.

### 2.4. Molecular Docking Studies 

**Figure 2:**
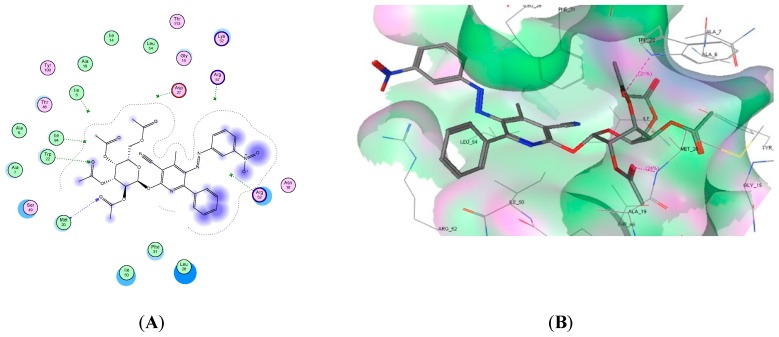
(**A**) 2D interactions of compound **8_d_** with the DHFR binding site. (**B**) 3D interactions of compound **8_d_** with the DHFR binding site.

**Figure 3:**
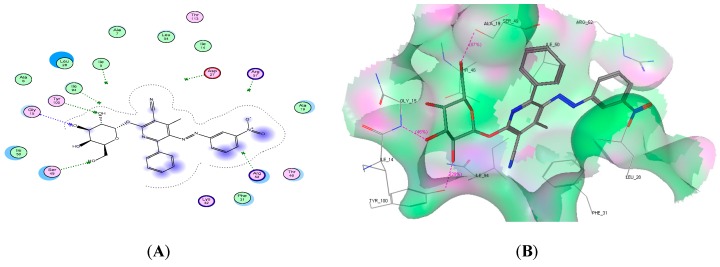
(**A**) 2D interactions of compound **9_d_** with the DHFR binding site. (**B**) 3D interactions of compound **9_d_** with the DHFR binding site.

**Figure 4:**
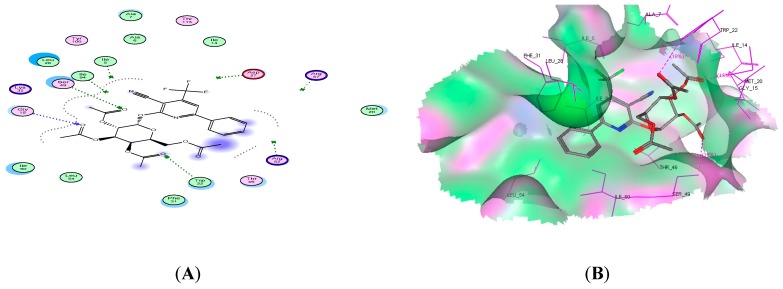
(**A**) 2D interactions of compound **11_c_** with the DHFR binding site. (**B**) 3D interactions of compound **11_c_** with the DHFR binding site.

**Figure 5:**
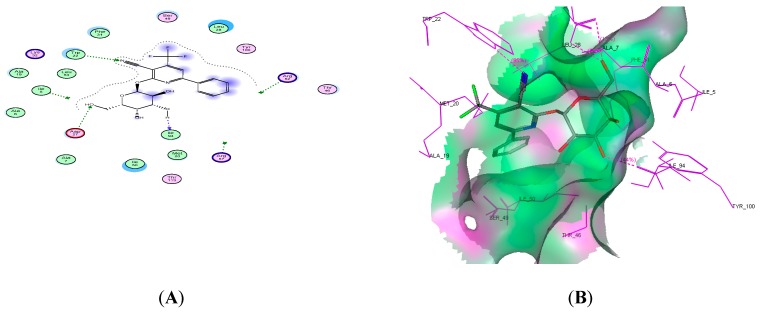
(**A**) 2D interactions of compound **14_c_** with the DHFR binding site. (**B**) 3D interactions of compound **14_c_** with the DHFR binding site.

**Figure 6:**
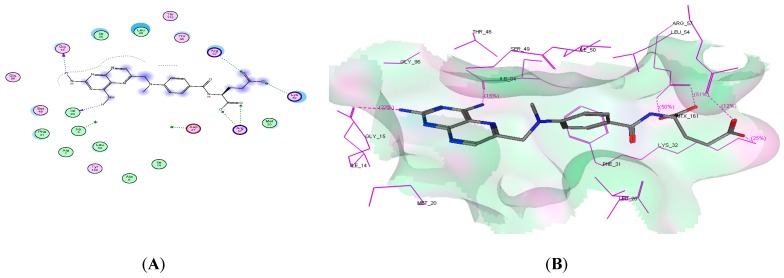
(**A**) 2D interactions of methotrexate with the DHFR binding site. (**B**) 3D interactions of methotrexate with the DHFR binding site.

Molecular docking is one of the most preferred methods in structure-based drug design, as it gives a good exploration into the binding mode of the new small molecules in the binding site of their appropriate targets. Understanding binding behavior is a key step in rational drug design [21,22]. Docking studies were performed in this research work to give insight into possible interactions, the docking score, and the mode of binding between the enzyme active binding site and the new bioactive molecules. Dihydrofolate reductase is a well-known target for infectious diseases, and DHFR inhibitors represent an important class of chemotherapeutic agents, as this enzyme is a key enzyme in the synthesis of thymidylate, and therefore DNA. Compounds that inhibit the DHFR enzyme have been found to have antibiotic properties. This enzyme is also considered to be a primary target for the development of new anticancer agents [23]. Thus, finding a new generation of DHFR inhibitors will be so useful in producing new anti-infective agents. The newly synthesized compounds **8_d_**, **9_d_**, **11_c_**, and **14_c_** were revealed to be more potent than penicillin against *S. aureus*, as shown in Figure 1. In addition, compounds **8_d_**, **9_d_**, **11_c_**, and **14_c_** showed good anticancer activity. Consequently, it is thought worldwide that docking studies are beneficial in supporting in vitro activity. The previously mentioned active compounds were chosen for docking using the Molecular Operating Environment (MOE) software program [24] to find the potential of these molecules against the DHFR enzyme and also to know the hypothetical binding mode of these active compounds in the active site of the dihydrofolate reductase enzyme. The crystal structure of the DHFR enzyme in complex with its inhibitor methotrexate was obtained from (PDB:ID:4DFR) [25]. Methotrexate was extracted and redocked into the active site of DHFR to perform validation, and then it was replaced with the new compounds in order to compare the binding mode of the tested compounds to methotrexate. All of the docked compounds showed comparable docking scores with methotrexate, and some of them showed lower docking energy as compound **8_d_**. The amino acid residue revealed that compounds **12_d_** and **13_c_** shared the Gly15 amino acid as methotrexate, and compound **14_c_** formed a hydrogen bond with the lle94 amino acid as methotrexate. In addition, compounds **13_c_** and **14_c_** formed three hydrogen bonds with the binding site. The obtained binding interactions and docking scores are represented in Table 5, and also 2D and 3D interactions for all of the docked compounds and methotrexate are pictured in Figure 2, Figure 3, Figure 4, Figure 5 and Figure 6.

## 3. Conclusion

Green synthesis techniques were employed to synthesize 2-pyridone galactoside analogues (**8_a–d_** and **11_a–c_**) at a high yield within a very short time. Here, 2D-NMR revealed clear structure elucidation for the resulting compounds. Antimicrobial screening showed that some of the obtained derivatives had potential activity against the growth of both G+ and G- tested bacteria. The new derivatives **9_c_**, **9_d_**, **11_c_**, and **14_c_** were revealed to be more potent than penicillin against *S. aureus*. In addition, compounds **8_d_**, **9_d_**, and **14_c_** showed moderate anticancer activity. Moreover, molecular docking studies revealed the good binding affinity of these molecules with the DHFR binding site, which was comparable to that of methotrexate.

## 4. Materials and Methods

### 4.1. Chemistry

General: A microwave synthetic protocol was performed using a CEM Microwave system. Melting points were determined on (Pyrex capillary) a Gallenkamp apparatus. Infrared spectra were recorded with a Thermo Nicolet Nexus 470 FT-IR spectrometer (Thermo Scientific, Waltham, MA, USA) in the range 4000–400 cm^−1^ in potassium bromide disks. Ultraviolet absorption spectra in the region 200–600 nm were recorded on a Secoman Anthelie 2 Advanced spectrophotometer (Secomam, France) in 1.00-cm cells at 25 °C. The spectra were run in spectra quality methanol using a concentration of 5 × 10^−5^ M. ^1^H-NMR spectra, 1D-NMR spectra, and 2D-NMR (COSY, NOESY, Ghmbc, and Ghmqc) spectra were obtained on a Varian Gemini 400-MHz FT NMR spectrometer (Varian, Agilent Technologies, Edinburgh, UK) in CDCl_3_ and DMSO-*d*_6_. Chemical shifts were recorded in (ppm) units relative to Me_4_Si as an internal standard. The mass spectra were recorded on a Shimadzu LCMS-QP 800 LC-MS (Shimadzu, Tokyo, Japan) and an AB-4000 Q-trap LC-MS/MS (Applied Biosystems, Grand Island, NY, USA). Analytical data were obtained using a PerkinElmer 2400 II series CHN Analyzer (Perkin Elmer, Waltham, MA, USA). Optical rotations were measured with a PerkinElmer digital polarimeter (Perkinelmer, Waltham, MA, USA) at 589 nm (sodium D line) in a 1-dm cell. Thin-layer chromatography (TLC) was carried out on precoated Merck silica gel F_254_ plates, and UV light was used for visualization. Column chromatography was performed on Merck silica gel. The reagents were purchased from Aldrich (St. Louis, MO, USA) and were used without further purification. 

#### 4.1.1. General Procedure for the Synthesis of 3-Cyano-2-(2″,3″,4″,6″-tetra-O-acetyl-β-d-galactopyranosyloxo)-pyridines (**8_a–d_**, **11_a–c_**)

##### Microwave Methods A (Solvent-Free Method)

Pyridine-2(1*H*)-ones (**3_a–d_, 10_a–c_**) (10 mmol) were mixed with 1″,2″,3″,4″,6″-penta-*O*-acetyl-α-d-galacto-pyranose **9** (11 mmol, 4.29 g). A mixture was dissolved of methylene chloride/methanol (80/20) followed by the addition of 1.0 g of silica gel (200–400 mesh), and then the solvent was removed by evaporation. The dried residue was transferred into a 10-mL vial and irradiated for 2–3 min using a CEM Microwave system. The product was purified using column chromatography in MeOH (0–2%) CHCl_3_ to afford the products (**8_a–d_**, **11_a–c_**).

##### Microwave Methods B (Catalyst-Free Method)

Pyridinium salts (**4_a–d_, 13_a–c_**) (10 mmol) were dissolved in acetone or a mixture of acetone/DMF (5 mL, 10 mmol), and 2″,3″,4″,6″-tetra-*O*-acetyl-α-d-galactopyranosyl bromide **7** (11 mmol, 4.52 g) was added. The reaction mixture was irradiated for a suitable time using a CEM Microwave system. The reaction mixture was filtered off, and the solvent was evaporated under reduced pressure at room temperature. The product was dried and purified using column chromatography in MeOH (0–2%) CHCl_3_ to afford the products (**8_a–d_**, **11_a–c_**). 

##### Conventional Synthesis Method C (Silyl Method)

Pyridine-2(1*H*)-ones (**3_a–d_, 10_a–c_**) (10 mmol) were heated under reflux, with stirring and under anhydrous conditions, for 48 h with hexamethyldisilazane (25 mL) and (NH_4_)_2_SO_4_ (0.02 g). The excess of the HMDS was removed under diminished pressure, providing the silylated bases as colorless oils. To a solution of silylated base in dry MeCN (30 mL) was added a solution of 1″,2″,3″,4″,6″-penta-*O*-acetyl-α-d-galactopyranose (**6)** (11 mmol, 4.29 g) in dry MeCN (10 mL), followed by SnCl_4_ (1.6 mL). The reaction mixture was stirred at room temperature until the reaction was judged complete through TLC. The reaction mixture was poured into saturated NaHCO_3_ solution and extracted with CHCl_3_ (3 × 20 mL). The organic layers were dried over MgSO_4_, filtered, and concentrated to give the crude nucleoside. The crude product was dried and purified using column chromatography in MeOH (0–2%) CHCl_3_ to afford the products (**8_a–d_**, **11_a–c_**).

3-Cyano-4,6-dimethyl-2-(2″,3″,4″,6″-tetra-*O*-acetyl-β-d-galactoyranosyl-oxy)-5-(4′-chlorophenylazo)-pyridine (**8_a_**): mp 189 °C; IR (KBr, cm^−1^) υ 2227 (CN), 1755 (CO); COSY; NOESY; gHMBC; ^1^H-NMR (400 MHz, CDCl_3_) δ = 2.05, 2.055, 2.060, and 2.07 (4s, 12H, 4CH_3_CO), 2.61 (s, 3H, CH_3_), 2.62 (s, 3H, CH_3_), 3.95–4.00 (m, 1H, H-5″), 4.17 (t, 1H, H-4″, *J_H-4__″-H-3__″_* = 9.2 Hz), 4.22 (t, 1H, H-3″, *J_H-3__″-H-4__″_* = 9.2 Hz), 5.23 (t, 1H, H-2″, *J_H-2__″-H-1__″_* = 7.8 Hz), 5.32–5.37 (m, 2H, H-6″), 6.01 (d, 1H, H-1″, *J* = 8.2 Hz,), 7.63 (d, 2H, Ar-H, *J* = 8.6 Hz), 7.71 (d, 2H, Ar-H, *J* = 8.6 Hz); ^13^C-NMR (100 MHz, CDCl_3_) δ = 18.1 (CH_3_), 19.5, 19.55, 20.0, and 20.5 (4CH_3_CO), 22.3 (CH_3_), 61.2 (C-6″), 67.5 (C-5″), 70.4 (C-4″), 72.5 (C-3″), 72.7 (C-2″), 94.1(C-3), 97.1 (C-1″), 112.7 (CN), 123.8–154.7 (Ar-C), 159.6 (C-2), 168.5, 168.8, 169.2, and 170.1 (4CO); LC-MS (ionization method): *m*/*z* 618 [M + 1]; Anal. calcd for C_28_H_29_ClN_4_O_10_: C, 54.51; H, 4.74; N, 9.08%. Found C, 54.61; H, 4.67; N, 9.22%.

3-Cyano-4,6-dimethyl-2-(2″,3″,4″,6″-tetra-*O*-acetyl-β-d-galactopyranosylox-y)-5-(3^′^-nitrophenylazo) (**8_b_**): mp 218 °C; IR (KBr, cm^−1^) υ 2228 (CN), 1753 (CO); COSY; NOESY; gHMBC; ^1^H-NMR (400 MHz, CDCl_3_) δ = 1.95, 2.00, 2.05, and 2.06 (4s, 12H, 4CH_3_CO), 2.58 (s, 3H, CH_3_), 2.61 (s, 3H, CH_3_), 3.93–3.98 (m, 1H, H-5″), 4.18 (t, 1H, H-4″, *J_H-4__″-H-3__″_* = 9.2 Hz), 4.25 (t, 1H, H-3″, *J_H-3__″-H-4__″_* = 9.2 Hz), 5.21 (t, 1H, H-2″, *J_H-2__″-H-1__″_* = 7.8 Hz), 5.34–5.38 (m, 2H, H-6″), 5.97 (d, 1H, H-1″, *J* = 8.1 Hz), 7.65–7.80 (m, 4H, Ar-H); ^13^C-NMR (100 MHz, CDCl_3_) δ = 18.1 (CH_3_), 19.5, 19.55, 20.0, and 20.5 (4CH_3_CO), 22.3 (CH_3_), 61.2 (C-6″), 67.5 (C-5″), 70.4 (C-4″), 72.5 (C-3″), 72.7 (C-2″), 94.1(C-3), 97.1 (C-1″), 112.7 (CN), 123.8–154.7 (Ar-C), 159.6 (C-2), 168.5, 168.8, 169.2 and 170.1 (4CO); LC-MS (ionization method): *m*/*z* 627 [M]; Anal. calcd for C_28_H_29_N_5_O_12_: C, 53.59; H, 4.66; N, 11.16%. Found: C, 53.71; H, 4.82; N, 10.95%. 

3-Cyano-4-methyl-2-(2″,3″,4″,6″-tetra-*O*-acetyl-β-d-galactoyranosyloxy)-6-phenyl-5-(4′-chlorophenylazo)-pyridine (**8_c_**): mp 178 °C; IR (KBr, cm^−1^) υ 2228 (CN), 1757 (CO); COSY; NOESY; gHMBC; ^1^H-NMR (400 MHz, CDCl_3_) = 1.97, 1.99, 2.01, and 2.02 (4s, 12H, 4CH_3_CO), 2.56 (s, 3H, CH_3_), 3.91-3.97 (m, 1H, H-5″), 4.20 (t, 1H, H-4″, *J_H-4__″-H-3__″_* = 9.2 Hz), 4.26 (t, 1H, H-3″, *J_H-3__″-H-4__″_* = 9.2 Hz), 5.23 (t, 1H, H-2″, *J_H-2__″-H-1__″_* = 7.8 Hz), 5.32–5.37 (m, 2H, H-6″), 5.99 (d, 1H, H-1″, *J* = 8.1 Hz), 7.33–7.81 (m, 9H, Ar-H); ^13^C-NMR (100 MHz, CDCl_3_) δ = 18.7 (CH_3_), 20.6, 20.65, 20.7, and 20.8 (4CH_3_CO), 62.3 (C-6″), 68.4 (C-5″), 70.5 (C-4″), 72.9 (C-3″), 73.1 (C-2″), 94.8 (C-3), 98.1 (C-1″), 113.8 (CN), 124.2–154.7 (Ar-C), 159.7 (C-2), 168.3, 169.6, 170.0 and 170.9 (4CO); LC-MS (ionization method): *m*/*z* 679 [M + 1]; Anal. calcd for C_33_H_31_ClN_4_O_10_: C, 58.37; H, 4.60; N, 8.25%. Found: C, 58.61; H, 4.73; N, 8.11%. 

3-Cyano-4-methyl-2-(2″,3″,4″,6″-tetra-*O*-acetyl-β-d-galactopyranosyloxy)-6-phenyl-5-(3^′^-nitrophenylazo)-pyridine (**8_d_**): mp 168 °C; IR (KBr, cm^−1^) υ 2230 (CN), 1756 (CO); COSY; NOESY; (400 MHz, CDCl_3_) = 1.95, 2.01, 2.07, and 2.09 (4s, 12H, 4CH_3_CO), 2.60 (s, 3H, CH_3_), 3.99–4.03 (m, 1H, H-5″), 4.22 (t, 1H, H-4″, *J_H-4__″-H-3__″_* = 9.2 Hz), 4.29 (t, 1H, H-3″, *J_H-3__″-H-4__″_* = 9.2 Hz), 5.33 (t, 1H, H-2″, *J_H-2__″-H-1__″_* = 7.8 Hz), 5.37–5.40 (m, 2H, H-6″), 6.09 (d, 1H, H-1″, *J* = 8.0 Hz), 7.33–7.81 (m, 9H, Ar-H); ^13^C-NMR (100 MHz, CDCl_3_) δ = 18.7 (CH_3_), 20.5, 20.55, 20.6, and 20.7 (4CH_3_CO), 61.8 (C-6″), 68.4 (C-5″), 70.5 (C-4″), 72.3 (C-3″), 73.2 (C-2″), 94.8 (C-3), 97.9 (C-1″), 113.5 (CN), 123.2–154.7 (Ar-C), 160.2 (C-2), 168.8, 169.5, 170.3 and 170.5 (4CO); LC-MS (ionization method): *m*/*z* 690 [M + 1]; Anal. calcd for C_33_H_31_N_5_O_12_: C, 57.47; H, 4.53; N, 10.16%. Found: C, 57.58; H, 4.62; N, 10.33%. 

3-Cyano-4,6-dimethyl-2-(2″,3″,4″,6″-tetra-*O*-acetyl-β-d-galactopyranosylox-y) pyridine (**11_a_)**: mp 147 °C; IR (KBr, cm^−1^) υ 2232 (CN), 1758 (CO); ^1^H-NMR (400 MHz, CDCl_3_) δ = 2.01, 2.01, 2.03, and 2.05 (4s, 12H, 4CH_3_CO), 2.43 (s, 3H, CH_3_), 2.52 (s, 3H, CH_3_), 4.02–4.05 (m, 1H, H-5″), 4.24 (t, 1H, H-4″, *J_H-4__″-H-3__″_* = 9.2 Hz), 4.31 (t, 1H, H-3″, *J_H-3__″-H-4__″_* = 9.2 Hz), 5.31 (t, 1H, H-2″, *J_H-2__″-H-1__″_* = 7.8 Hz), 5.39–5.44 (m, 2H, H-6″), 6.02 (d, 1H, H-1″, *J_H-1__″-H-2__″_* = 8.0 Hz), 6.80 (s, 1H, pyridine H-5); ^13^C-NMR (100 MHz, CDCl_3_) δ = 20.4 (CH_3_), 20.6, 20.7, 20.75, and 20.8 (4CH_3_CO), 24.3 (CH_3_), 61.3 (C-6″), 67.8 (C-5″), 70.7 (C-4″), 72.8 (C-3″), 74.1 (C-2″), 93.8 (C-3), 95.0 (C-1″), 114.2 (CN), 120.2 (C-5), 154.8 (C-4), 160.8 (C-6), 161.7 (C-2), 169.1, 170.0, 170.5 and 170.8 (4CO); LC-MS (ionization method): *m*/*z* 479 [M + 1]; Anal. calcd for C_22_H_26_N_2_O_10_: C, 55.23; H, 5.48; N, 5.86%. Found: C, 55.46; H, 5.72; N, 5.98%. 

3-Cyano-4-methyl-2-(2″,3″,4″,6″-tetra-*O*-acetyl-β-d-galactopyranosyloxy)-6-phenyl-pyridine (**11_b_**): mp 167 °C; IR (KBr, cm^−1^) υ 2230 (CN), 1764 (CO); ^1^H-NMR (400 MHz, CDCl_3_) δ = 2.00, 2.01, 2.02, and 2.03 (4s, 12H, 4CH_3_CO), 2.57 (s, 3H, CH_3_), 3.90–3.94 (m, 1H, H-5″), 4.12 (t, 1H, H-4″, *J_H-4__″-H-3__″_* = 9.2 Hz), 4.11 (t, 1H, H-3″, *J_H-3__″-H-4__″_* = 9.2 Hz), 5.22 (t, 1H, H-2″, *J_H-2__″-H-1__″_* = 7.8 Hz), 5.40-5.46 (m, 2H, H-6″), 5.97 (d, 1H, H-1″, *J_H-1__″-H-2__″_* = 8.0 Hz), 7.40 (s, 1H, pyridine H-5), 7.39–7.45 (m, 3H, Ar-H), 7.94–7.99 (m, 2H, Ar-H); ^13^C-NMR (100 MHz, CDCl_3_) δ = 20.4, 20.5, 20.6, and 20.7 (4CH_3_CO), 24.2 (CH_3_), 62.4 (C-6″), 68.6 (C-5″), 70.5 (C-4″), 72.8 (C-3″), 73.1 (C-2″), 93.7 (C-3), 96.3 (C-1″), 113.8 (CN), 116.3 (C-5), 127.2–137.1 (Ar-C), 155.6 (C-4), 157.5 (C-6), 161.4 (C-2), 168.3, 168.9, 169.8, and 170.8 (4CO); LC-MS (ionization method): *m*/*z* 563 [M + Na]^+^; Anal. calcd for C_27_H_28_N_2_O_10_: C, 60.00; H, 5.22; N, 5.18%. Found: C, 59.88; H, 5.42; N, 5.30%.

3-Cyano-2-(2″,3″,4″,6″-tetra-*O*-acetyl-β-d-galactopyranosyloxy)-4-trifluromethyl-6-phenyl-pyridine (**11_c_**): mp 161 °C; IR (KBr, cm^−1^) υ 2230 (CN), 1753 (CO); ^1^H-NMR (400 MHz, CDCl_3_) δ = 2.00, 2.01, 2.02, and 2.03 (4s, 12H, 4 CH_3_CO), 3.97–4.01 (m, 1 H, H-5″), 4.19 (t, 1H, H-4″, *J_H-4__″-H-3__″_* = 9.2 Hz), 4.08 (t, 1H, H-3″, *J_H-3__″-H-4__″_* = 9.2 Hz), 5.19 (t, 1H, H-2″, *J_H-2__″-H-1__″_* = 7.8 Hz), 5.42-5.47 (m, 2H, H-6″), 6.12 (d, 1H, H-1″, *J_H-1__″-H-2__″_* = 8.0 Hz), 7.51–7.53 (m, 3H, Ar-H), 7.68 (s, 1H, pyridine H-5), 7.84–8.03 (m, 2H, Ar-H); ^19^F-NMR (376 MHz, CDCl_3_) δ = (-63.81) (s, 3F, CF_3_); ^13^C-NMR (100 MHz, CDCl_3_) δ = 20.0, 20.1, 20.15 and 20.2 (4CH_3_CO), 61.8 (C-6″), 68.0 (C-5″), 70.1 (C-4″), 72.3 (C-3″), 73.0 (C-2″), 91.8 (C-1″), 94.3 (C-3), 110.7 (CN), 110.9 (C-5), 119.8 (CF_3_), 127.2–135.2 (Ar-C), 144.9 (C-4), 160.0 (C-6), 162.2 (C-2), 168.3, 168.8, 169.3, and 170.4 (4 CO); LC-MS (ionization method): *m*/*z* 595 [M + 1]; Anal. calcd for C_27_H_25_F_3_N_2_O_10_: C, 54.55; H, 4.24; N, 4.71%. Found: C, 54.76; H, 4.51; N, 4.52%.

#### 4.1.2. General Procedure for the Synthesis of 3-cyano-2-(β-d-galactopyranosyloxy)-pyridines (**9_a–d_**, **14_a–c_**).

##### General Procedure for Nucleoside Deactylation Method A

Triethylamine (1.0 mL) was added to a solution of protected galactosides (**11_a–d_, 13_a–c_**) (10 mmol) in 10 mL MeOH and 3 drops of water. The mixture was stirred for 18 h at room temperature. The solvent was evaporated under reduced pressure, and the residue was coevaporated with MeOH until triethylamine was removed. The residue was crystallized from an appropriate solvent to give deprotected galactosides (**9_a–d_, 14_a–c_**).

##### General Procedure for Nucleoside Deactylation Method B

Dry ammonia gas was passed into a solution of protected galactosides (**8_a–d_, 11_a–c_**) (0.5 g) in 20 mL of dry methanol at 0 °C for 30 min. The reaction mixture was stirred until completion, as shown by TLC (10–12 h), using chloroform/methanol 19:1 (R_f_ 0.60–62 region). The resulting mixture was then concentrated under reduced pressure to afford a crude solid. The crude products were purified by silica gel chromatography (chloroform/methanol 20:1). The products were crystallized from methanol to furnish the products (**9_a–d_, 14_a–c_**).

3-Cyano-4,6-dimethyl-2-(β-d-galactopyranosyloxy)-5-(4′-chlorophenylazo)-pyridine (**9_a_**): mp 144 °C; IR (KBr, cm^−1^) υ 3431 (OH), 2227 (CN); ^1^H-NMR (400 MHz, DMSO-*d*_6_) δ = 2.44 (s, 3H, CH_3_), 2.63 (s, 3H, CH_3_), 3.28-3.71 (m, 6H, H-2″, H-3″, H-4″, H-5″, 2H-6″), 4.62–5.59 (4OH, exchangeable with D_2_O), 5.8 (d, 1H, H-1″, *J_H-1__″-H-2__″_* = 7.7 Hz), 7.71–7.82 (m, 4H, Ar-H); ^13^C-NMR (100MHz, DMSO-*d*_6_) δ = 19.5 (CH_3_), 24.4 (CH_3_), 60.6 (C-6″), 68.8 (C-5″), 72.8 (C-4″), 76.7 (C-3″), 77.6 (C-2″), 96.8 (C-3), 98.2 (C-1″), 114.6 (CN), 125.2–156.8 (Ar-C), 160.8 (C-2); LC-MS (ionization method): *m*/*z* 448 [M]; Anal. calcd for C_20_H_21_ClN_4_O_6_: C, 53.52; H, 4.72; Cl, 7.90; N, 12.48%. Found: C, 53.71; H, 4.93; N, 12.76%.

3-Cyano-4,6-dimethyl-2-(β-d-galactopyranosyloxy)-5-(3^′^-nitrophenylazo)-pyridine (**9_b_**): mp 147 °C; IR (KBr, cm^−1^) υ 3421 (OH), 22,230 (CN); ^1^H-NMR (400 MHz, DMSO- *d*_6_) δ = 2.44 (s, 3 H, CH_3_), 2.63 (s, 3H, CH_3_), 3.31–3.77 (m, 6 H, H-2″, H-3″, H-4″, H-5″, 2H-6″), 4.71–5.60 (4OH, exchangeable with D_2_O), 5.91 (d, 1 H, H-1″, *J_H-1__″-H-2__″_* = 7.8 Hz), 7.61–7.89 (m, 4H, Ar-H); ^13^C-NMR (100 MHz, DMSO-*d*_6_) δ = 18.1 (CH_3_), 22.5 (CH_3_), 60.2 (C-6″), 68.7 (C-5″), 72.8 (C-4″), 76.7 (C-3″), 77.6 (C-2″), 96.2 (C-1″), 97.2 (C-3), 114.1 (CN), 122.3-153.9 (Ar-C), 160.6 (C-2); LC-MS (ionization method): *m*/*z* 460 [M + H]; Anal. calcd for C_20_H_21_N_5_O_8_: C, 52.29; H, 4.61; N, 15.24%. Found: C, 52.48; H, 4.87; N, 14.93%. 

3-Cyano-4-methyl-2-(β-d-galactopyranosyloxy)-6-phenyl-5-(4′-chlorophen-ylazo)-pyridine (**9_c_**): mp 157 °C; IR (KBr, cm^−1^) υ 3418 (OH), 2227 (CN); ^1^H-NMR (400 MHz, DMSO-*d*_6_) δ = 2.61 (s, 3H, CH_3_), 3.23-3.80 (m, 6H, H-2″, H-3″, H-4″, H-5″, 2H-6″), 4.77–5.83 (4OH, exchangeable with D_2_O), 5.91 (d, 1H, H-1″, *J_H-1__″-H-2__″_* = 7.2 Hz), 7.45–7.87 (m, 9H, Ar-H); ^13^C-NMR (50 MHz, DMSO-*d*_6_) δ = 18.1 (CH_3_), 60.6 (C-6″), 69.6 (C-5″), 72.6 (C-4″), 76.8 (C-3″), 77.8 (C-2″), 96.4 (C-1), 96.8 (C-3), 114.1 (CN), 123.8–153.6 (Ar-C), 160.5 (C-2); LC-MS (ionization method): *m*/*z* 511 [M + 1]; Anal. calcd for C_25_H_23_ClN_4_O_6_: C, 58.77; H, 4.54; Cl, 6.94; N, 10.97%. Found: C, 58.61; H, 4.83; N, 10.72%.

3-Cyano-4-methyl-2-(β-d-galactopyranosyloxy)-6-phenyl-5-(3^′^-nitrophenyl-azo)pyridine (**9_d_**): mp 136 °C; IR (KBr, cm^−1^) υ 3390 (OH), 2231 (CN); ^1^H-NMR (400 MHz, DMSO-*d*_6_) δ = 2.67 (s, 3H, CH_3_), 3.11–3.76 (m, 6 H, H-2″, H-3″, H-4″, H-5″, 2H-6″), 4.67–5.49 (4OH, exchangeable with D_2_O), 5.89 (d, 1H, H-1″, *J_H-1__″-H-2__″_* = 7.5 Hz), 7.41–7.75 (m, 9H, Ar-H); ^13^C-NMR (100 MHz, DMSO-*d*_6_) δ = 18.7 (CH_3_), 60.5 (C-6″), 69.9 (C-5″), 72.7 (C-4″), 76.8 (C-3″), 77.5 (C-2″), 96.7 (C-3), 97.1 (C-1″), 114.1(CN), 122.0–153.1 (Ar-C), 159.8 (C-2); LC-MS (ionization method): *m*/*z* 522 [M + 1]; Anal. calcd for C_25_H_23_N_5_O_8_: C, 57.58; H, 4.45; N, 13.43%. Found: C, 57.37; H, 4.67; N, 13.62%.

3-Cyano-4,6-dimethyl-2-(β-d-galactopyranosyloxy)-pyridine (**14_a_**): mp 201 °C; IR (KBr, cm^−1^) υ 3422 (OH), 2227 (CN); ^1^H-NMR (400 MHz, DMSO- *d*_6_) δ = 2.56 (s, 3H, CH_3_), 2.61 (s, 3H, CH_3_), 3.32–3.78 (m, 6H, H-2″, H-3″, H-4″, H-5″, 2H-6″), 4.65–5.46 (4OH, exchangeable with D_2_O), 5.91 (d, 1H, H-1″, *J_H-1__″-H-2__″_* = 7.9 Hz), 7.09 (s, 1H, pyridine H-5); ^13^C-NMR (100 MHz, DMSO-*d*_6_) δ = 20.1 (CH_3_), 24.3 (CH_3_), 60.7 (C-6″), 70.2 (C-5″), 73.1(C-4″), 76.9 (C-3″), 77.2 (C-2″), 93.2 (C-3), 96.3 (C-1″), 114.2 (CN), 119.2 (C-5), 155.2 (C-4), 161.0 (C-6), 161.9 (C-2); Anal. calcd for C_14_H_18_N_2_O_6_: C, 54.19; H, 5.85; N, 9.03%. Found: C, 54.03; H, 5.83; N, 9.12%.

3-Cyano-4-methyl-2-(β-d-galactopyranosyloxy)-6-phenyl-5-pyridine (**14_b_**): mp 212 °C; IR (KBr, cm^−1^) υ 3391 (OH), 2224 (CN); ^1^H-NMR (200 MHz, DMSO-*d*_6_) δ = 2.50 (s, 3H, CH_3_), 3.24–3.65 (m, 6H, H-2″, H-3″, H-4″, H-5″, 2H-6″), 4.57–5.37 (4OH, exchangeable with D_2_O), 6.08 (d, 1H, H-1″, *J_H-1__″-H-2__″_* = 6.6 Hz), 7.50–7.52 (m, 3H, Ar-H), 7.79 (s, 1H, pyridine H-5), 8.11–8.13 (m, 2H, Ar-H);^13^C-NMR (50 MHz, DMSO-*d*_6_) δ = 20.2 (CH_3_), 60.5 (C-6″), 69.5 (C-5″), 72.7 (C-4″), 76.9 (C-3″), 77.9 (C-2″), 94.6 (C-1″), 96.3 (C-3), 114.3 (CN), 115.4 (C-5), 127.0–136.2 (Ar-C), 155.8 (C-4), 156.3 (C-6), 161.8 (C-2); Anal. calcd for C_19_H_20_N_2_O_6_: C, 61.29; H, 5.41; N, 7.52%. Found: C, 61.20; H, 5.56; N, 7.70%.

3-Cyano-2-(β-d-galactopyranosyloxy)-4-trifluromethyl-6-phenyl-pyridine (**14_c_**): mp 142 °C; IR (KBr, cm^−1^) υ 3418 (OH), 2233 (CN); ^1^H-NMR (400 MHz, DMSO-*d*_6_) δ = 3.35–3.49 (m, 6H, H-2″, H-3″, H-4″, H-5″, 2H-6″), 4.62–5.51 (4OH, exchangeable with D_2_O), 5.97 (d, 1 H, H-1″, *J_H-1__″-H-2__″_* = 7.7 Hz), 7.58–7.60 (m, 3H, Ar-H), 8.31 (s, 1H, pyridine H-5), 8.31–8.34 (m, 2H, Ar-H); ^19^F-NMR (376 MHz, DMSO-*d*_6_) δ = (−62.71) (s, 3F, CF_3_); ^13^C-NMR (100 MHz, DMSO-*d*_6_) δ = 60.8 (C-6”), 69.1 (C-5″), 72.8 (C-4″), 76.8 (C-3″), 77.8 (C-2″), 90.2(C-3), 97.2 (C-1″), 112.0 (C-5), 113.5 (CN), 118.8 (CF_3_), 128.0–136.5 (Ar-C), 143.3 (C-4), 160.8 (C-6), 162.3 (C-2); LC-MS (ionization method): *m*/*z* 427 [M + 1]; Anal. calcd for C_19_H_17_F_3_N_2_O_6_: C, 53.53; H, 4.02; N, 6.57%. Found: C, 53.67; H, 4.22; N, 6.70%.

### 4.2. Biological Activity Evaluations

#### 4.2.1. Antimicrobial Activity

##### Materials and Methods

The synthesized compounds (**8_a–d,_ 9_a–d,_ 11_a–c_**, and **14_a–c_**) were tested for their in vitro growth inhibitory activity against a panel of standard strains of the Institute of Fermentation of Osaka (IFO, Osaka, Japan), namely gram-positive bacteria (*Staphylococcus aureus* IFO 3060 and *Bacillus subtilis* IFO 3007) and gram-negative bacteria (*Escherichia coli* IFO 3301 and *Proteus vulgaris* IFO 3851). The primary screening was carried out using the agar disc-diffusion method using Müller–Hinton agar medium [26].

##### Agar Disc-Diffusion Method

Sterile filter paper discs (5 mm in diameter) were moistened with the compound solution in dimethylsulfoxide of a specific concentration (30 µg/disc) and were carefully placed on the agar culture plates, which had been previously inoculated separately with the microorganisms. The plates were incubated at 37 °C for 24 h, and the diameter of the clear zone of inhibition surrounding the sample was taken as a measure of the inhibitory power of the sample against the particular test organism (% inhibition = sample inhibition zone (cm)/plate diameter × 100). All measurements were done in DMSO as a solvent, which has zero inhibition activity. The obtained results were compared to some reference antibiotics. 

#### 4.2.2. Anticancer Activity

##### Materials and Methods: Cell Culture and Viability Assay

Human breast cancer cells (MDA-MB-231) and liver cancer cells (HT29) (Caliper LifeSciences, Waltham, MA, USA) were maintained in DMEM medium (Invitrogen, Cergy Pontoise, France), and human lung cancer cells (NCI-H460-Luc2) (Caliper LifeSciences) (NSCLC) were maintained in RPMI 1640 (Hyclone Thermo Scientific, MA, USA). All media were supplemented with antibiotics (penicillin 50 U/mL; streptomycin 50 mg/mL) (Invitrogen, Cergy Pontoise, France) and 10% fetal bovine serum (FBS, Biowest, Nouaille, France). Cells were seeded in 96-well plates at a density of 5000 cells/well. After 24 h, the cells were treated for 24 h or 48 h with increasing concentrations of the test compounds (5–100 µM) in triplicate. Control cultures were treated with 0.1% DMSO. The effect of the test compounds on cell viability was determined using a CellTiter-Glo Luminescent Cell Viability assay (Promega Corporation, Madison, WI, USA) based on a quantification of ATP, which signals the presence of metabolically active cells. The luminescent signal was measured using a GLOMAX Luminometer system. The data are presented as a proportional viability (%) from comparing the treated group to the untreated cells, the viability of which was assumed to be 100% [27].

#### 4.2.3. Molecular Docking

The molecular docking studies were performed using the Molecular Operating Environment (MOE; Chemical Computing Group, Montreal, QC, Canada). Energy minimizations were performed at an RMSD gradient value of 0.05 kcal/mol Å and an MMFF94X force field, and partial charges were automatically added. The methotrexate bound to dihydrofolate reductase (DHFR) enzyme (Pdb ID:4DFR) was obtained from the Protein Data Bank. The cocrystallized ligand with the enzyme was checked and refined, and hydrogen atoms were added: Water molecules and bound ligands were removed. The active site was detected using the MOE-Alpha site finder. A validation process was performed by redocking the cocrystallized ligand in the DHFR binding site. The selected compounds were drawn in 2D form, saved as mol, and then transformed into 3D using the MOE program: The energy was minimized, protonated, and then saved as a data base file for docking. Docking for the selected compounds was applied, and amino acid interactions, the scoring energy, and hydrogen bonds were determined. 

## Figures and Tables

**Table 1:** Yield and reaction time comparison between microwave and conventional methods in the synthesis of 5-arylazo-3-cyano-2-(2″,3″,4″,6″-tetra-*O*-acetyl-β-d-galactopyranosyloxo)-pyridines (**8_a–d_**).

Compound No.	R	Ar	Microwave Synthesis		Conventional Synthesis
			Reaction Time/min Yield (%)		Reaction Time/h Yield (%)
			Method A	Method B	Method C
**8_a_**	CH_3_	4-ClC_6_H_4_	2 (92)	5 (86)	53 (50)
**8_b_**	CH_3_	3-NO_2_C_6_H_4_	3 (90)	5 (81)	53 (49)
**8_c_**	C_6_H_5_	4-ClC_6_H_4_	2 (95)	4 (85)	51 (44)
**8_d_**	C_6_H_5_	3-NO_2_C_6_H_4_	2 (93)	4 (83)	51 (51)

**Table 2:** Yield and reaction time comparison between conventional and microwave-assisted synthesis of 3-Cyano-2-(2″,3″,4″,6″-tetra-*O*-acetyl-β-d-galactopyranosyloxo)-pyridines (**11_a–c_**).

Compound No.	R^1^	R^2^	Microwave Synthesis		Conventional Synthesis
			Reaction Time/min Yield (%)		Reaction Time/h Yield (%)
			Method A	Method B	Method C
**11_a_**	CH_3_	CH_3_	3 (87)	7 (80)	62 (61)
**11_b_**	C_6_H_5_	CH_3_	2 (91)	7 (83)	67 (70)
**11_c_**	C_6_H_5_	CF_3_	2 (94)	6 (85)	65 (61)

**Table 3:** Yield comparison of triethylamine and ammonia methods for synthesis of deprotected galactosides (**9_a–d_**).

Comound No.	R	Ar	Method A Yield %	Method B Yield %
**9_a_**	CH_3_	4-ClC_6_H_4_	90	83
**9_b_**	CH_3_	3-NO_2_C_6_H_4_	88	87
**9_c_**	C_6_H_5_	4-ClC_6_H_4_	87	83
**9_d_**	C_6_H_5_	3-NO_2_C_6_H_4_	89	80

**Table 4:** Yield comparison of triethylamine and ammonia methods for synthesis of deprotected galactosides (**14_a–c_**).

Comound No.	R^1^	R^2^	Method A Yield %	Method B Yield %
**14_a_**	CH_3_	CH_3_	85	81
**14_b_**	C_6_H_5_	CH_3_	87	81
**14_c_**	C_6_H_5_	CF_3_	90	83

**Table 5:** The docking scores and binding interactions of the docked compounds inside the DHFR active site.

Compound No.	Docking Score (kcal/mol)	No. of H-Bonds	Amino Acid Residue	Interacting Group
**8_d_**	−21.86	2	Met20 and Trp22	2 C=O
**9_d_**	−19.91	3	Gly15, Ser49, and Tyr100	3 OH
**11_c_**	−19.09	3	Ser49, Tre22, and Gly15	3 C=O
**14_c_**	−19.59	3	Trp22, Asp27, and lle94	CN, 2OH
**Ligand**	−19.54	6	Gly15, lle94, Arg57, Lys32, and Arg52	2NH_2_ and 2 COO
